# Electroencephalographic biomarkers of cognitive impairment in bipolar disorder

**DOI:** 10.1192/j.eurpsy.2026.11608

**Published:** 2026-07-31

**Authors:** N. Smaoui, H. Sghaier, L. Triki, I. Gassara, R. Feki, M. Maalej, N. Charfi, M. Maalej, S. Omri, L. Zouari

**Affiliations:** 1 Psychiatry “C” , Hedi Chaker University Hospital; 2 Department of Functional Explorations, Habib Bourguiba University Hospital, Sfax, Tunisia

## Abstract

**Introduction:**

Impaired cognitive function is common in patients with bipolar disorder (BD), and its pathophysiology has been the subject of multiple theories. Research has aimed to identify neurophysiological biomarkers that reflect cognitive impairment in euthymic bipolar patients.

**Objectives:**

To identify electroencephalographic specificities of cognitive impairment by analyzing electroencephalogram (EEG) tracings in patients with bipolar disorder type I (TB-1), compared with control subjects, that may serve as biomarkers of cognitive impairment in TB.

**Methods:**

This was a cross-sectional, case-control study. It was carried out at the “C” psychiatry outpatient unit at Hedi Chaker University Hospital, Sfax, Tunisia. Fifteen TB-1 patients and fifteen healthy controls were included. All participants were assessed by the Screen For Cognitive Impairment in Psychiatry (SCIP) scale for evaluation of cognitive functions. They underwent standard waking EEG with eyes closed at the Department of Functional Explorations, Habib Bourguiba Hospital, Sfax, Tunisia. Each participant was given a mental arithmetic test during the EEG, and absolute power spectral density (PSD) analysis was performed to compare brain activity at rest and after cognitive stimulation.

**Results:**

Cognitive assessment from EEG data revealed significant differences between bipolar patients and healthy controls. At rest, a notable distinction was observed in beta1 band PSD between right and left temporal regions in bipolar patients, but this difference attenuated after mental exercise. In addition, bipolar patients showed significantly lower PSD values in temporal regions, both at rest and after cognitive stimulation, compared with controls. Linear regression analysis revealed negative correlations between SCIP total score and alpha1 wave PSD in the left frontal and right temporal regions, as well as positive correlations with alpha1 wave PSD in the right frontal region and alpha2 wave in the left temporal region.

**Conclusions:**

This study highlighted significant alterations in electroencephalographic patterns in patients with TB-1. The differences observed in PSD, particularly in the alpha and beta bands, could serve as potential biomarkers for assessing cognitive deficits in TB-1.

**Disclosure of Interest:**

None Declared

